# Heating-Rate-Triggered Carbon-Nanotube-based 3-Dimensional Conducting Networks for a Highly Sensitive Noncontact Sensing Device

**DOI:** 10.1038/srep19632

**Published:** 2016-01-28

**Authors:** Yanlong Tai, Gilles Lubineau

**Affiliations:** 1 King Abdullah University of Science and Technology (KAUST), Physical Sciences and Engineering Division, COHMAS Laboratory, Thuwal 23955-6900, Saudi Arabia

## Abstract

Recently, flexible and transparent conductive films (TCFs) are drawing more attention for their central role in future applications of flexible electronics. Here, we report the controllable fabrication of TCFs for moisture-sensing applications based on heating-rate-triggered, 3-dimensional porous conducting networks through drop casting lithography of single-walled carbon nanotube (SWCNT)/poly(3,4-ethylenedioxythiophene)-polystyrene sulfonate (PEDOT:PSS) ink. How ink formula and baking conditions influence the self-assembled microstructure of the TCFs is discussed. The sensor presents high-performance properties, including a reasonable sheet resistance (2.1 kohm/sq), a high visible-range transmittance (>69%, PET = 90%), and good stability when subjected to cyclic loading (>1000 cycles, better than indium tin oxide film) during processing, when formulation parameters are well optimized (weight ratio of SWCNT to PEDOT:PSS: 1:0.5, SWCNT concentration: 0.3 mg/ml, and heating rate: 36 °C/minute). Moreover, the benefits of these kinds of TCFs were verified through a fully transparent, highly sensitive, rapid response, noncontact moisture-sensing device (5 × 5 sensing pixels).

Flexible and transparent electronics (FTEs) will be integral to the next generation of electronics, for which classical silicon electronics are not suitable^1^,^2^,^3^. Instead, transparent conductive films (TCFs) on plastic substrates are likely to be the essential components^4^. TCFs based on indium tin oxide (ITO) have already been successfully applied in portable, wearable, and attachable electronic systems, such as organic solar cells^5^, organic light-emitting diodes^6^, organic photodetectors^7^, liquid-crystal displays^8^, and touch screen films^9^. However, on flexible substrates, indium tin oxide (ITO) film performance is limited for flexible electronics because as the film mechanically degrades, their conductivity quickly decreases. This technology is also challenged by a limited reserve of indium and a complex manufacturing process^10^,^11^.

These limitations have motivated efforts towards developing alternative materials for TCFs, including new transparent conducting oxides^12^, metallic nanowires^13^, carbon-based materials (e.g., nanotubes and graphene)^14^,^15^, conductive polymers^16^, ultrathin metal films^17^, and patterned conductive grids or rings^18^ and their hybrid composites^19^. They all not only exhibit high transparency across the visible light spectrum (Transmittance, up to 90%) and low sheet resistance (below 10 ohm/sq), but they also present excellent dynamic fatigue properties (above 1000 cycles)^17^. In particular, carbon-based TCFs are affordable and show high stability upon exposure to high temperatures and ultraviolet light^11^.

Recently, interest towards improving carbon-based 3-dimensional (3D) porous conducting networks (3D-PCNs) is growing^20^. 3D microstructures have the potential to increase surface area, offer unusual or novel physical and electronic properties, and extend unsurpassed chemical functionality^21^. These features are attractive for application to nanoelectronics^22^, energy storage/conversion^23^, catalysis^24^, gas/humidity sensing^25^, and water collecting^26^. To date, these 3D-PCNs have predominantly been fabricated by the following dominant processes: etching with a high concentration of waste lye^27^, blocking copolymers using the template method with plenty of polymer residue^28^, or using the self-assembly method using a toxic nonpolar solvent like benzene or chloroform^29^. Drawbacks for each of these processes have inspired the development of a more facile and effective method of fabrication.

Here, we develop single-walled carbon-nanotube-based porous transparent conductive films (SWCNT-PTCFs) with self-assembled 3-dimensional conducting networks using a facile and novel approach.

First, SWCNTs with high aspect ratio (2500–20000) were chosen as material for the conductive backbone. To inhibit the agglomeration of SWCNTs, poly(3,4-ethylenedioxythiophene)/polystyrene sulfonate (PEDOT:PSS) was used as a dispersant because of the π–π intermolecular interaction between SWCNTs and conjugated thiophene chains in PEDOT:PSS, confirmed by our previous research^30^. We prepared stable and monodisperse SWCNT/PEDOT:PSS inks, which we display along with their TEM images in Fig. 1a and Fig. S1. The advantages of using PEDOT:PSS as a dispersing agent is that it creates a highly conductive interface at CNT/CNT junctions. We used this strategy in our previous work to design highly conductive thermoplastic composites enriched with PEDOT:PSS-coated CNTs^30^.

Second, SWCNT-PTCFs were fabricated by the drop casting method (Fig. 1b). Ink properties and baking processes, such as the ratio between SWCNT and PEDOT:PSS, the concentration of SWCNT, and heating rate, were investigated systematically. Our objective was to promote a transition from a 2-dimensional (2D) to a 3D microstructure of the conducting networks by increasing the heating rate throughout the baking process, allowing the fabrication of SWCNT-PTCFs to be controlled (Fig. 1c). Note that the fabrication process will be largely dependent on both electrical and mechanical behaviors of PEDOT:PSS with humidity, and chaotic accumulation of the high aspect ratio and flexibility of SWCNT^31^,^32^. Porous graphene films were prepared alongside for comparison.

Third, the efficiency of fabricated SWCNT-PTCFs on polyethylene terephthalate (PET) was measured by assessing the physical properties of the resulting sensor. Properties were investigated using Raman spectra, sheet resistance, dynamic fatigue properties, and total optical/diffuse transmittance (T_T_ and T_D_) with a haze value (=T_D_/T_T_). Taken together, these results provided detailed information on the physical properties of SWCNT-PTCFs, which are useful for determining where they can be suitably applied.

Finally, we demonstrate the practical benefits of SWCNT-PTCFs by applying them to a fully transparent, highly sensitive, rapid response, noncontact moisture sensing film with 5 × 5 sensing pixels. Here, SWCNT-PTCFs are used to detect the proximity of a human index finger without direct contact based only on detecting humidity. The high performance of SWCNT-PTCFs was proven by their high sensitivity, good stability, and rapid response/recovery ability. In the future, this technology could have potential for a different sort of human–machine interaction, compared to existing touch panels that operate through direct contact. And lastly, we demonstrated the sensitivity of the film by tracking the real-time movement of an insect in 3D space (e.g., an ant).

## Results

### Characterization of SWCNT/PEDOT:PSS ink

The quality of SWCNTs, and the homogeneity and stability of its ink are integral to the final SWCNT-based porous transparent conductive film product. Hence, SWCNTs with an outer diameter of 1–2 nm and a length of 5–30 μm were chosen for the transparent conductive material, as shown in Fig. 2a. The high aspect ratio of these CNTs (up to 2500–30000) makes them excellent candidates to effectively create a continuous and steady conductive network at a low percolation threshold value.

**Figure 1:**
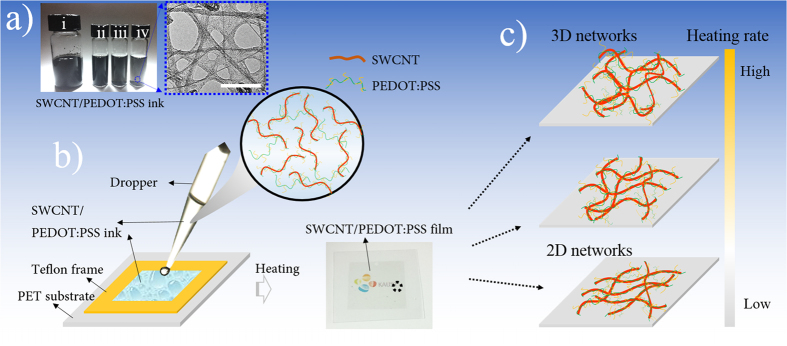
(**a**) Digital image of SWCNT/PEDOT:PSS inks with different concentrations ((i) 12.5 mg/ml, (ii) 0.3 mg/ml, (iii) 0.2 mg/ml, (iv); 0.1 mg/ml) and its TEM image (the scale bar is 50 nm); (**b**) Schematic illustration of the preparation of SWCNT-PTCFs; (**c**) Schematic illustration of the transition from a 2D to a 3D microstructure with varying heating rates.

After surface modification via PEDOT:PSS, the resulting inks of different concentrations were very stable for at least 1 month without apparent precipitation, as shown in Fig. 1a, also confirmed by TEM images in Fig. S1. Each SWCNT can be identified with clear boundaries. Moreover, a very thin and unsmooth PEDOT/PSS layer (approximately 1–4-nm thick or thicker due to a hydrophilic amorphous phase from PSS) on the outer wall of SWCNTs can be observed, indicating that the agglomeration of SWCNTs was successfully prevented. This can be attributed to the conjugated aromatic chains from PEDOT:PSS that can strongly anchor to the surface of SWCNT via π–π interactions without disrupting the electronic structure of the SWCNT^30^.

### Preparation of SWCNT-PTCFs

**Figure 2:**
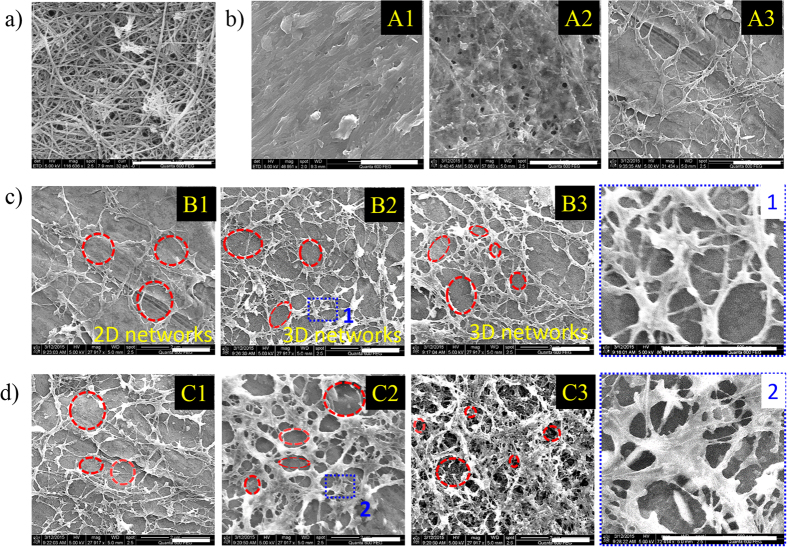
SEM images of SWCNT-PTCFs with self-assembled 3D porous microstructures at different magnifications. (**a**) Pure SWCNTs; (**b**) Different ratios of SWCNT to PEDOT:PSS (A1: 1:1, A2: 1:0.75, and A3: 1.0.5, respectively); (**c**) Different heating rates (B1: 0 °C/minutes, B2: 36 °C/minutes, and B3: 72 °C/minutes, respectively); Fig. 1 is a zoom on the blue-marked domain in B2 (**d**) Different concentrations of SWCNT (C1: 0.1 mg/ml, C2: 0.2 mg/ml, and C3: 0.3 mg/ml, respectively); Fig. 2 is a zoom on the blue-marked domain in C2. The default ratio of SWCNT to PEDOT:PSS, SWCNT concentration, and heating rate is 1:0.5, 0.1 mg/ml, and 36 °C/minute, respectively. All scale bars are 1 μm.

SWCNT-PTCF microstructures can be effectively controlled using different ink formulas and baking conditions, as shown in Fig. 2b–d.

The influence of the ratio between SWCNT and PEDOT:PSS in the ink formula is discussed in Fig. 2b. We found that when the ratio is 1:1, SWCNTs and PEDOT:PSS chains were completely intertwined, making it difficult to distinguish the existence of SWCNTs. When this ratio increases to 1:0.75, the crossed networks of SWCNTs become detectable, and porosities emerge with diameters in the 30–100-nm range. This phenomenon can be explained by the fact that PEDOT:PSS films between the crossed SWCNTs begin to shrink during the baking process. When this ratio is increased to 1:0.5, the amount of PEDOT:PSS becomes insufficient to prevent the PEDOT:PSS films between the crossed SWCNTs from completely breaking apart during the baking process. As a result, a highly porous network of SWCNTs is formed, which is only slightly attached to the substrate and with much greater porosities in the 100-nm to 1000-nm range.

The influence of heating rate on the microstructure of SWCNT-PTCFs is summarized in Fig. 2c (B1, B2, B3, and Fig. 1C). The results show that as heating rate increases, the whole SWCNT-PEDOT/PSS assembly begins to form a 3D structure. More specifically, the porosities between the crossed SWCNTs emerge in the vertical and horizontal planes, gradually forming a 3D porous microstructure of TCFs with increased heating rate.

The formation of the porous structure in the horizontal plane comes from the break of PEDOT:PSS films among crossed SWCNTs and has little connection to heating rate; however, in the vertical plane, the porous structure is created by the volatilization of a solvent. At slower heating rates, SWCNTs coated with PEDOT:PSS have already deposited by gravity and adhered to firmly to the PET substrate before all of the solvent has volatilized: the result is a 2D TCF. Another distinct difference between 2D and 3D structures is that the SWCNT track in a 2D structure is wider than that in a 3D structure, due to the stack of SWCNTs coated with PEDOT:PSS in the vertical plane. As heating rate increases, the speed of volatilization of the solvent increases, leading to the drying of the PEDOT:PSS coat on SWCNTs before it has been completely deposited onto the PET substrate, which causes PEDOT:PSS film among the crossed SWCNTs to break, forming a porous structure in the vertical plane.

Most importantly, as we described earlier, SWCNTs with a high aspect ratio (up to 2500–30000) have excellent flexibility and strong spatial extension similar to carbon fibers, which helps maintain the stability of the 3D microstructure during the baking process. In addition, an earlier report noted that the physical properties of PEDOT:PSS have a high dependency on water content, which was attributed to water uptake during the hygroscopic (PSS-HSO_3_) phase that weakens the cohesion between PEDOT/PSS grains via hydrogen bonds. [30] Thus, during the baking process, the physical properties of PEDOT/PSS become more favorable as water content decreases, ultimately becoming glue-like bonds with SWCNT at junctions. For these reasons, a continuous and steadily conductive network with a 3D porous microstructure can be successfully fabricated.

Increasing the concentration of SWCNTs also caused the 3D film to form smaller holes (smaller than 30 nm in diameter) on the microstructure of the SWCNT-PTCFs, as shown in Fig. 2d (C1, C2, C3) due to the stack of SWCNTs, not only the thickness variation.

For comparison we also analyzed graphene/PEDOT:PSS film though graphene/PEDOT:PSS ink and found a strong relationship between the graphene microstructure and baking conditions with brightness variation of the film, as shown in Fig. S5. More specifically, brightness variation mainly results from the state-space transmission of graphene sheets, with a gradually increasing angle between the substrate with increased heating rate.

### Characterization of SWCNT-PTCFs

**Figure 3:**
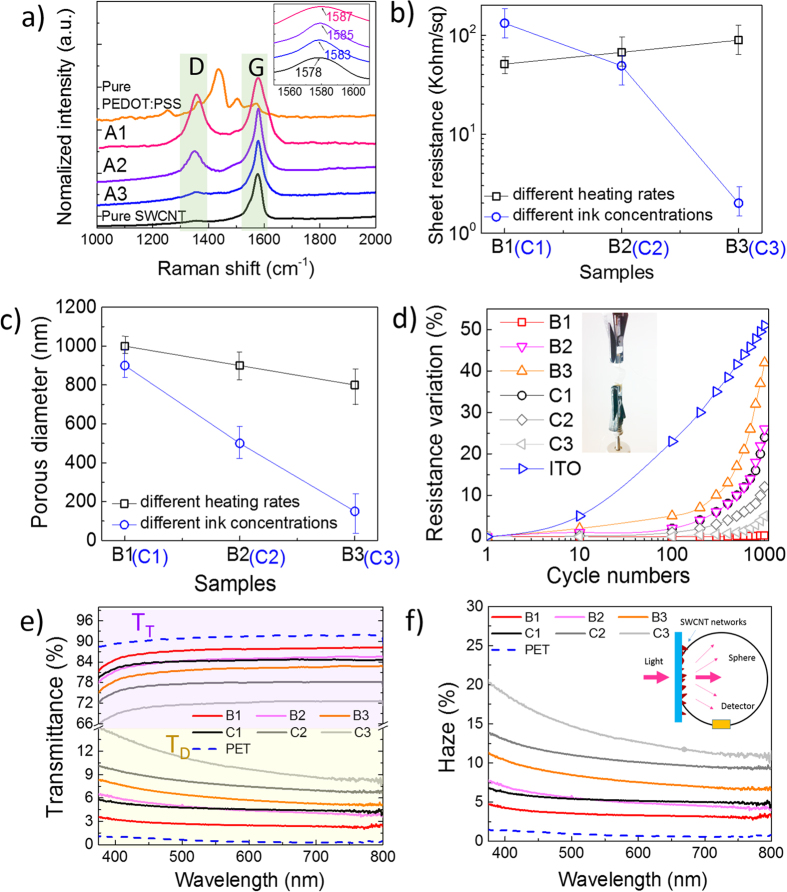
Performance of SWCNT-PTCFs with different microstructures. (**a**) Raman spectrum of pure SWCNT, pure PEDOT:PSS, and SWCNT-TCFs with different ratios (SWCNT to PEDOT:PSS, A1-1:1, A2-1:0.75, A3-1:0.5, respectively); the inset shows the shift of the G peak; (**b**) sheet resistance variation; (**c**) porous diameter variation; (**d**) mechanical properties; inset shows the test method (long cyclic tests under a dynamic force of 0.1 N with a preload of 0.1 N); and (**e**) Total optical/diffuse transmittance and (**f**) haze value variation. SWCNT-PTCFs with different heating rates were defined as B1 (0 °C/minute), B2 (36 °C/minute), and B3 (70 °C/minute); SWCNT-PTCFs with different ink concentrations were defined as C1 (0.1 mg/ml), C2 (0.2 mg/ml), and C3 (0.3 mg/ml).

To further explain the influence of different ratios of SWCNT to PEDOT:PSS, Raman spectrum were used to investigate the molecular changes in SWCNT-TCFs, as shown in Fig. 3a. A strong peak is evident at 1423 cm^−1^ in the spectra of pure PEDOT:PSS, which was assigned to symmetric *Cα* = *Cβ* stretching deformation in the aromatic thiophene ring^30^. Two characteristic modes (D and G) within 1000 - 2000 cm^−1^ were also distinguished in the spectra of pure SWCNTs. G mode denotes graphite mode, while D mode is present in all graphite-like carbons and originates from structural defects. The ratio between D and G is proportional to the sample’s purity^33^.

By comparing the spectrum, we see that the gradually increasing peak (D) at 1433 cm^−1^, clearly demonstrates the presence of PEDOT:PSS, and the D:G ratio increased with a higher content of PEDOT:PSS. Meanwhile, the red shift phenomena in mode G (from 1578 to 1583 to 1585 to 1587 cm^−1^) also can be found, confirming the π–π intermolecular interaction between SWCNTs and the conjugated thiophene chains in PEDOT:PSS. This interaction enhanced the phonon excitation energy with the increasing coherence length and electron mobility under electron-phonon scattering and binding^34^.

Figure 3b exhibits the electrical properties of the prepared SWCNT-PTCFs. With increasing SWCNT concentration from 0.1 mg/ml to 0.3 mg/ml, the sheet resistance of SWCNT-PTCFs decreases from 139 kohm/sq to 2.1 kohm/sq due to the increased thickness of SWCNT-PEDOT:PSS film and the decreased porous diameter from 800–1000 nm to 150–300 nm (Fig. 3c). Meanwhile, when the porous microstructure changed from 2D to 3D with increased heating rate, sheet resistance increased slightly from 51 kohm/sq to 89 kohm/sq. This is mainly because the porosity in the vertical plane decreased the stacking density of SWCNT along with decreased porous diameter from 900–1100 nm to 700–800 nm (Fig. 3c).

Mechanical flexibility and durability of the prepared SWCNT-PTCFs were also investigated in Fig. 3d. Variations in the mechanical-resistance of the films on the PET substrate were recorded as a function of cycle number under a fixed external compression stress. Meanwhile, the ITO film (130-nm thick) on the PET substrate was also constructed for comparison. Results show that sheet resistance increased gradually with a continuous compression cycle, especially for films with a 3D porous microstructure (incl. B1 to B3, C1 to C3) because they are less adhesive to the substrate. Adhesive ability increased with the increasing film thickness because, to a certain extent, thicker films inhibit the development of microscopic cracks that can form perpendicular to the compressing stress, as seen in the curves of C1 to C3. These results are favorable over those of ITO films with a drastic variation in resistance.

More details can be seen in Fig. S6. In general, our SWCNT-PTCFs exhibited excellent mechanical flexibility and durability under compression stress.

Figure 3e,f present the optical properties of the prepared SWCNT-PTCFs with PET film as a refs 35,36

All films exhibit stable and uniform total optical transmittance (T_T_) and diffuse transmittance (T_D_) in the visible range, and T_T_ is inversely proportional to the concentration of SWCNT and heating rate. T_T_ was expected to decrease with thicker films. The relationship with heating rate can be explained by the higher reflectivity of films with 3D networks compared with 2D networks. Finally, T_D_ increased with both the film thickness and heating rate. As a consequence, films obtained by high heating rate feature a higher haze that is a direct consequence of the 3D microstructure of the obtained network that is promoting light dispersion.

### Fully Transparent, flexible, noncontact sensing device

**Figure 4:**
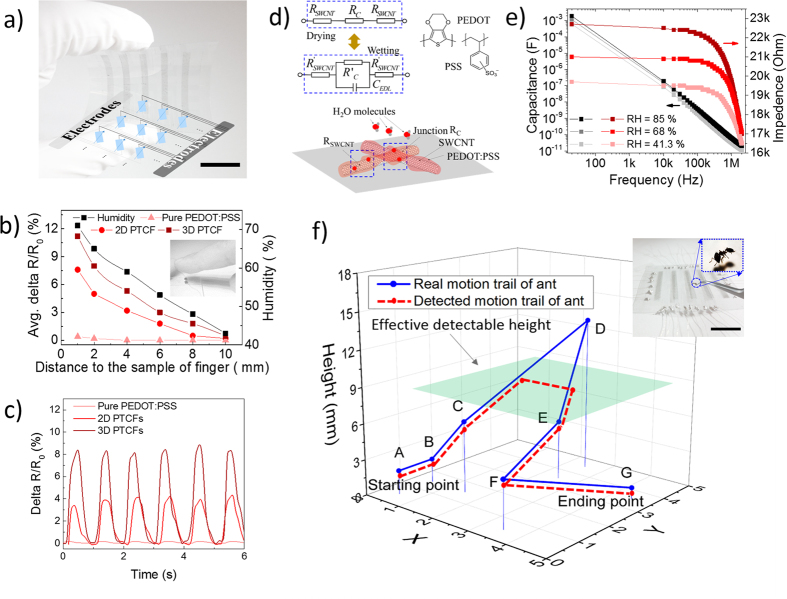
(**a**) Photograph of a typical flexible matrix noncontact sensing device (5 × 5 array) with the relevant schematic equivalent electric circuit, (**b**) Calibration of the relative humidity (RH) gradient of a human index finger with different distances by an RH meter and the resistance response of different SWCNT-PTCFs to a human index finger with different distances, (**c**) Short cyclic tests of the humidity response with different SWCNT-PTCFs, (**d**) Working mechanism of this noncontact sensing device, (**e**) Relationship between the capacitance/impedence of SWCNT-PTCFs and frequency under different RH gradients. (**f**) The trail of movement created by moving an ant with a pair of tweezers within a three-dimensional space through the fabricated sensing device to create a map; average ant size was 2.8 × 1 mm, and the green plane is the effective detectable height for the ant. All scale bars are 1 cm.

To demonstrate the benefit of the SWCNT-PTCFs, they were integrated into a fully transparent, flexible sensing panel with 5 × 5 sensing pixels. The fabrication process is described in the Experimental Section. The photograph and circuit schematic of this sensing device are shown in Fig. 4a.

To evaluate the sensitivity of the prepared SWCNT-PTCFs to relative humidity (RH) at room temperature, one of the sensing pixels from the above panel was selected and tested for its sensing response to a human index finger^14^,^37^,^38^.

In advance, the humidity field (to the distance) around the human index finger was demarcated via a humidity meter (TM325, Dickson), which is summarized in Fig. 4b. The RH decreased from 72.8 RH % to 43.2 RH % with an increasing distance from the meter of 1 mm to 1 cm, compared with the humidity of the room of 42.1 RH %. Results in Fig. 4b also show that the resistance response of the SWCNT-PTCFs was highly distance dependent due to the change in humidity. Specifically, sensitivity to humidity (defined as Delta R/R_0_) of PTCFs with a 3D microstructure is approximately 1.5-fold higher than that of PTCFs with a 2D microstructure; no change in the resistance response of PEDOT:PSS films was observed.

The real-time dynamic response curves of SWCNT-PTCFs with repeated distance (4 mm, to sensing films) of a human index finger were compared in Fig. 4c. Results indicate that these films can detect RH with high sensitivity, good stability, and fast response/recovery ability, reproducing with a stable signal output. The response and recovery time was defined as the time required to attaining 90% of the steady state value, which were typically only 0.5 s and 0.6 s, respectively, with the ratio of response time/recover time approaching 1 for PTCFs with 3D microstructure. These values were much better than those for PTCFs with a 2D microstructure, for PEDOT:PSS-TCFs, and than those previously reported^39^. These performances can be attributed to the 3D microstructure of PTCFs, which bare a greater surface area for exposure to ambient humidity.

For the working mechanism, according to previous description, there is a PEDOT:PSS layer coated on the outer wall of SWCNTs with the thickness of 1–4 nm through π–π interactions. This layer has strong humidity sensitivity because the hydrophilic amorphous phase from PSS can absorb water molecules, further generating hydronium ions (H_3_O^+^) or free hydrogen ions (H^+^), which form an electrical double layer (C_EDL_) between PEDOT:PSS chains, as shown in Fig. 4d and equation (1)^40^. This layer acts to reduce the conduction of original materials according to quantum conductance calculations^41^,^42^. More importantly, this phenomenon also take place at the junctions of SWCNTs, resulting in non-ohmic contacts, which is important in light of the 3D microstructure of PTCFs. These variations cause an increase in the total resistance.


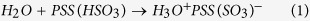


In addition, the dependence of the capacitance or impedance of 3D SWCNT-PTCFs to humidity and frequency was also investigated to verify the above explanation, as shown in Fig. 4e. The value of C_EDL_ is proportional to environment humidity: 189 nF (RH = 85%), 155 nF (RH = 68%), and 121 nF (RH = 41.3%) at 10 kHz Compared to 42 pF, 34 pF, and 29 pF at 2 MHz. The impedance value of the 3D SWCNT-PTCFs experiences a similar phenomenon. All of these results show that the existence of C_EDL_ changes with environmental humidity.

The high performance of our carbon-based humidity sensor reported here provides a basis for its future development towards a new age of noncontact human–machine interactions. We illustrated the benefits of our 3D SWCNT-PTCFs by continuously and accurately mapping the movement of an ant within a 3D space, as shown in Fig. 4e,f. Only point D (13.9 mm) was not accurately represented, but his was expected as this point fell outside of the defined space (7.6 mm, green plane in Fig. 4f). The location of the ant in X-Y direction was predicted by changes in the resistance of the sensing pixel, whereas the location of the ant in the Z direction can be estimated from the resistance variation of the sensing pixel according to the relationship between resistance variation and height. In this case no variation indicates that the ant is out of the defined space.

## Discussion

In conclusion, we have reported on the controllable fabrication of TCFs with a self-assembled 3D porous microstructure produced by drop casting SWCNT/PEDOT:PSS ink triggered by heating rate. We reviewed the roles of the ink formula and baking conditions on the microstructure of the SWCNT-PTCFs. Our SWCNT-PTCFs exhibited an acceptable level of electrical conductivity, a high visible-range transmittance, and an excellent dynamic mechanical property. The fully transparent, flexible, noncontact sensing device (5 × 5 sensing pixels) integrated with these SWCNT-PTCFs had high sensitivity, good stability, and rapid response/recovery ability; it could even continuously and accurately map the trail of a moving ant within a 3D space. Therefore, these types of films are promising for future application to noncontact human–machine interactions, for energy storage and conversion, gas adsorption and filtration, and as a catalyst because of its 3D porous microstructure.

## Methods

### Materials

The PEDOT/PSS aqueous dispersion (1.3 wt. %, Clevios™ PH1000) was purchased from HC Starck, Inc. Carboxyl group (−COOH) functionalized SWCNTs were purchased from CheapTubes, Inc.: tubes had an outer diameter of 1–2 nm, a length of 5–30 μm, over 95 wt. % purity, and 2.56 wt. % COOH groups. Graphene nanoplatelets were purchased from CheapTubes, Inc: tubes had a diameter of 2 μm, a surface area >700 m^2^/g, and over 99 wt. % purity. Teflon film tape (thickness = 0.08–013 mm) was purchased from Shaheen Enterprises. PET films were purchased from Teonex® Inc. with a thickness of 125 μm and 210 × 297 mm in size. ITO-coated PET films were purchased from Sigma-Aldrich (sheet resistance = 60 ohm/sq, PET thickness = 125 μm, ITO thickness = 130 nm, and transmittance >79% at 550 nm of wavelength). Deionized (DI) water was used in all experimental processes.

### Preparation of SWCNT/PEDOT:PSS ink and Graphene/PEDOT:PSS ink

SWCNT (0.25 g), PEDOT/PSS aqueous dispersions (PH1000) with different weight ratios (SWCNT to PEDOT:PSS) of 1:1, 1:0.75, 1:0.5, and DI water were combined in a glass bottle, and the solid content (12.5 mg/ml) of SWCNT was confirmed in the prepared suspensions. Next, the mixture was homogenized using a Brason 8510 bath sonicator (Thomas Scientific) for 1 h, followed by the exfoliation of the SWCNTs through an ultrasonic processor (Cole-Parmer) at 20 kHz and 500 W for 40 min in an ice bath to prevent extensive heating and damage to the SWCNTs and PEDOT:PSS. The inks with the desired concentrations (0.1 mg/ml, 0.2 mg/ml, and 0.3 mg/ml) were obtained by diluting the above mother inks with DI water. Typical inks are shown in Fig. 1a. A similar process was used to prepare graphene/PEDOT:PSS ink (0.1 mg/ml).

### Preparation of SWCNT/Graphene-PTCFs

SWCNT-PTCFs were prepared through the drop casting approach on a PET substrate, as shown in Fig. 1b. Specifically, PET films were treated with oxygen plasma at 100 W for 60 s as a substrate for better hydrophilicity. Next, we created a frame by attaching Teflon film tape with square holes (2.5 × 2.5 cm) to the substrate. The as-prepared SWCNT-PEDOT:PSS inks were dropped into the holes using a Thermo Scientific Finnpipette (0.2–2 μl) with a controlled concentration of 2 μl/cm^2^. Before the Teflon frame was peeled off, these inks were baked on a hotplate for 30 minutes with different heating rates (Fig. S2). Typical SWCNT-PTCFs are shown in Fig. S3. The whole process was performed gently to achieve a homogeneous film; graphene-PTCFs were prepared similarly.

### Fabrication of a transparent noncontact array sensor

First, a 5 × 5 ITO electrode array was patterned onto a PET film by shadow mask lithography and treated with oxygen plasma at 100 W for 60 s, and Teflon film tape with square holes (2 × 2 mm) was attached onto the film as a frame. Second, the as-prepared SWCNT/PEDOT:PSS inks were dropped into the holes using a Thermo Scientific Finnpipette (0.2–2 μl) with a controlled concentration of 2 μl/cm^2^. Before the Teflon frame was peeled off, these inks were baked on a hotplate for 30 minutes at different heating rates. Finally, copper wires were bonded to the surface of each ITO electrode using silver paste that is then cured on a hot plate at 100 °C for 1 h. More details are presented in Fig. S4.

### Characterization and measurements

The prepared SWCNT/PEDOT:PSS inks were examined by transmission electron microscopy (TEM, Titan G2 80–300 CT, FEI company) at an accelerating voltage of 300 kV to assess the quality of the SWCNT dispersion. Raman spectra of the surface of SWCNT-PTCFs were collected using a LabRAM Aramis Raman spectrometer (Horiba, Ltd.) on casted films using a 473-nm laser for structural analysis. Morphologies were investigated using scanning electron microscopy (SEM, Quanta 600, FEI Company). Sheet resistance was measured using a 4-point probe system (Pro4-440N, Lucas Labs). An ultraviolet-vis spectrometer (Cary 5000, Varian) with an integrated sphere was used to measure total and diffuse optical transmittance to evaluate the haze value. Finally, a PC-controlled universal test machine (Instron 5944 with a 5-N load cell) with a PC-recordable multimeter (Agilent 34401A) was used to perform the cyclic mechanical tests.

The fully transparent, flexible, noncontact sensor was characterized through a PC-recordable multimeter (Agilent 34401A) for mapping the real-time distance-resistance response. A human index finger and an ant were used as humidity source samples, and the humidity around each was defined through a humidity meter (TM325, Dickson).

## Additional Information

**How to cite this article**: Tai, Y. and Lubineau, G. Heating-Rate-Triggered Carbon-Nanotube-based 3-Dimensional Conducting Networks for a Highly Sensitive Noncontact Sensing Device. *Sci. Rep.* **6**, 19632; doi: 10.1038/srep19632 (2016).

## Supplementary Material

Supplementary Information
