# Supplementary material for: Heating-Rate-Triggered Carbon-Nanotube-based 3-Dimensional Conducting Networks for a Highly Sensitive Noncontact Sensing Device
Source: Sci Rep. 2016 Jan 28;6:19632. doi: 10.1038/srep19632 (PMC4730204; doi:10.1038/srep19632)
Supplement: Supplementary Information [file srep19632-s1.pdf]

Supporting information for

Heating-Rate-Triggered Carbon-Nanotube-based 3-Dimensional Conducting Networks for a Highly Sensitive Noncontact Sensing Device

Yanlong Tai, Gilles Lubineau*

King Abdullah University of Science and Technology (KAUST), Physical Sciences and Engineering Division, COHMAS Laboratory, Thuwal 23955-6900, Saudi Arabia

*Corresponding author

E-mail: yanlong.tai@kaust.edu.sa (Yanlong Tai), gilles.lubineau@kaust.edu.sa (Gilles Lubineau)

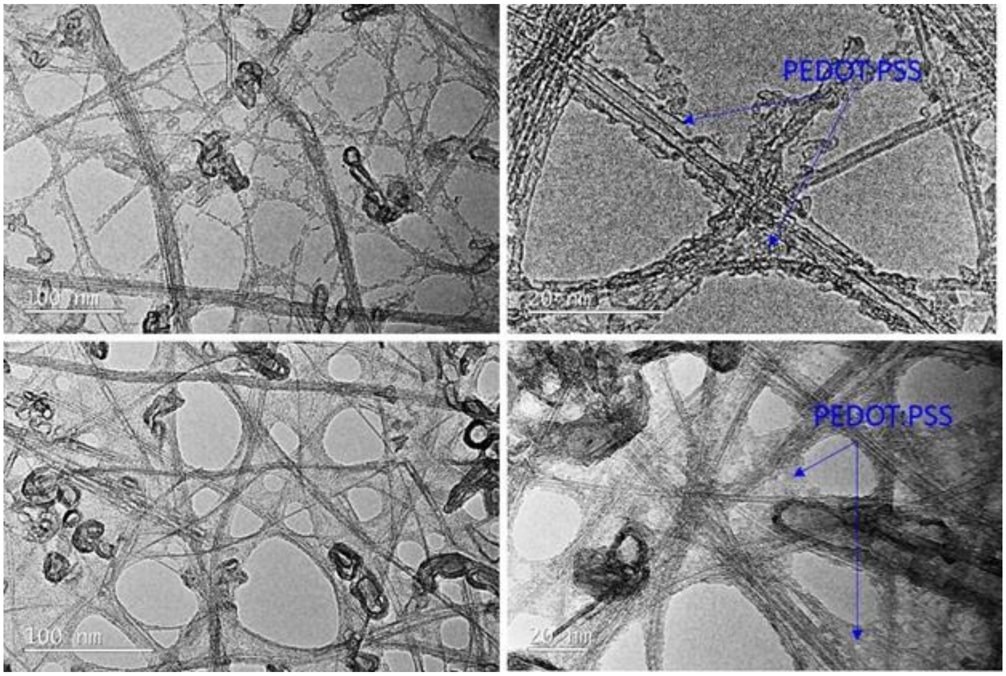

Figure S1. TEM of SWCNT/PEDOT:PSS ink (diluted concentration) at different magnifications

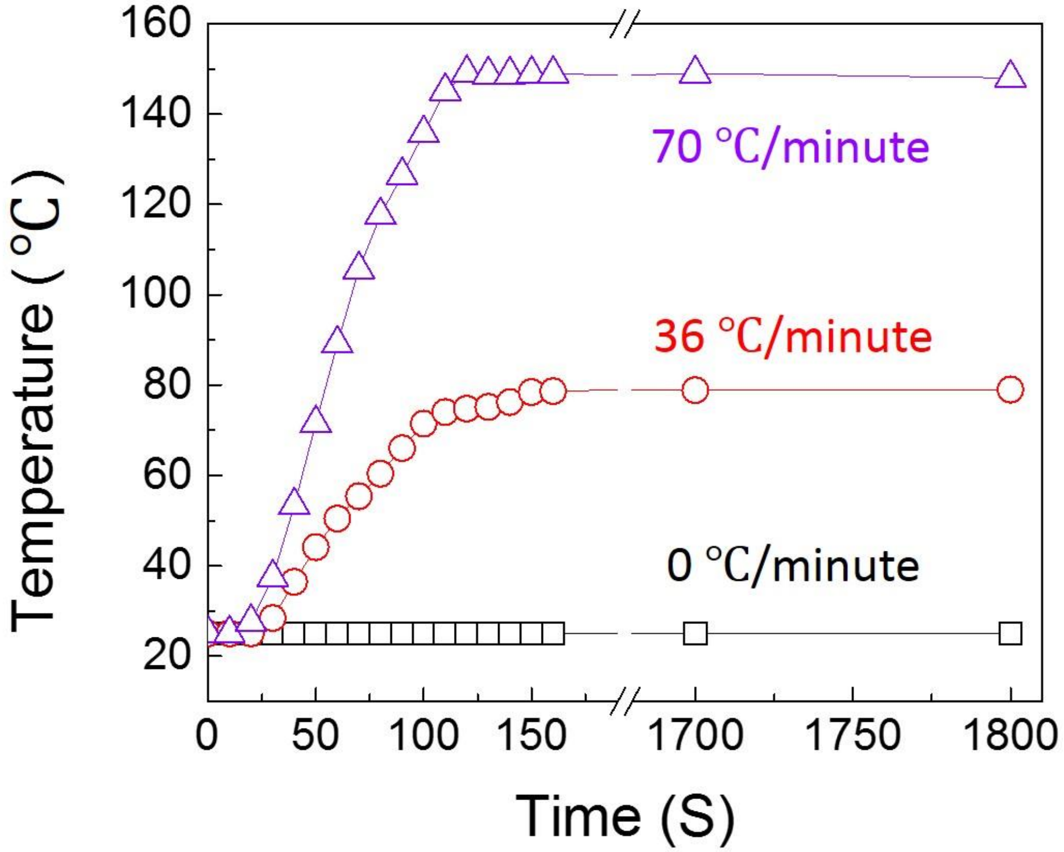

Figure S2. Heating rate during the fabrication of SWCNT-PTCFs (S1).

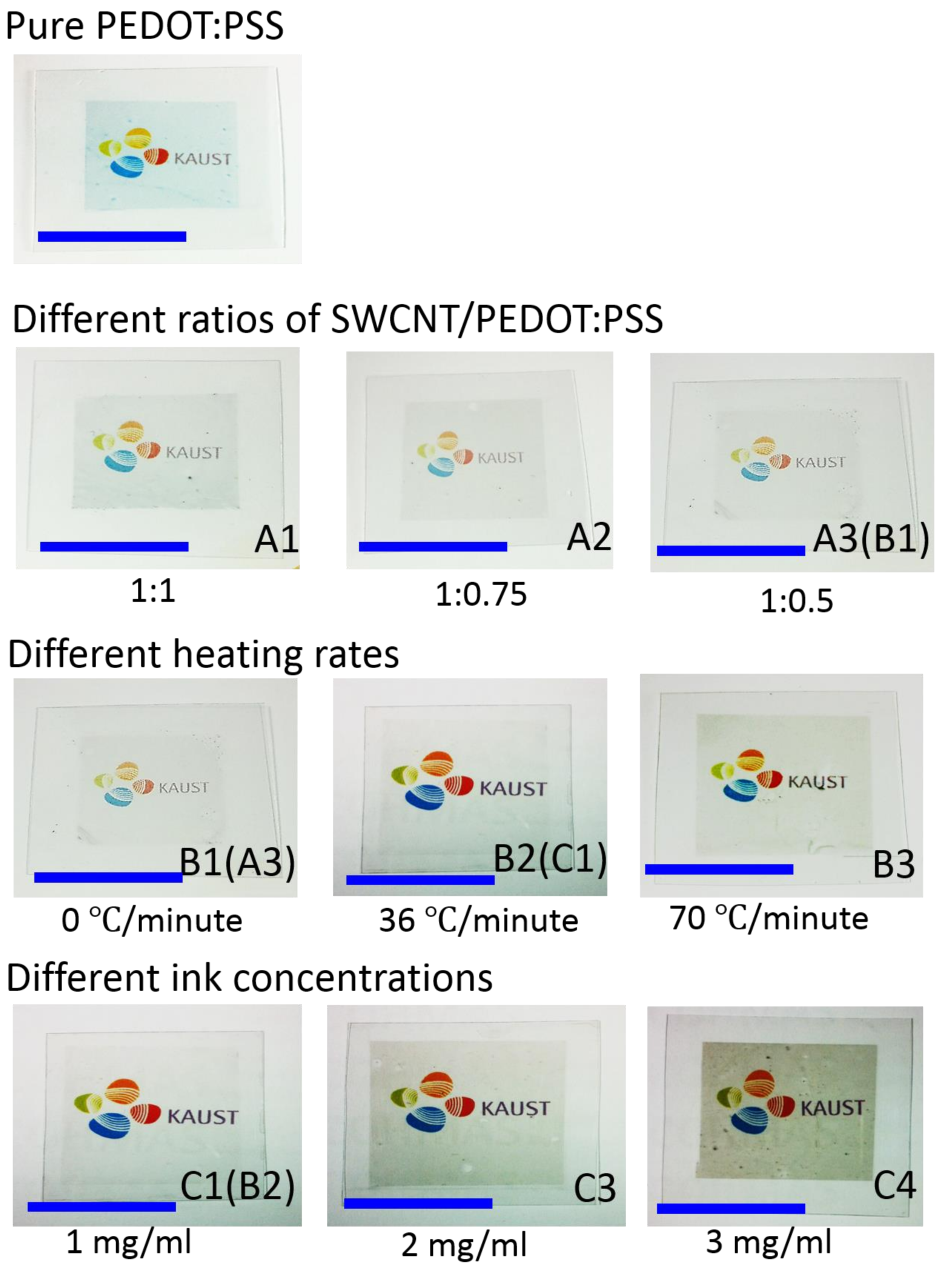

Figure S3. Typical SWCNT-TCFs under different fabrication conditions. The default ratio of SWCNT vs. PEDOT:PSS, SWCNT concentration, and heating rate is 1:0.5, 1 mg/ml, and 36 °C/minute, respectively. All scale bars are 1 cm.

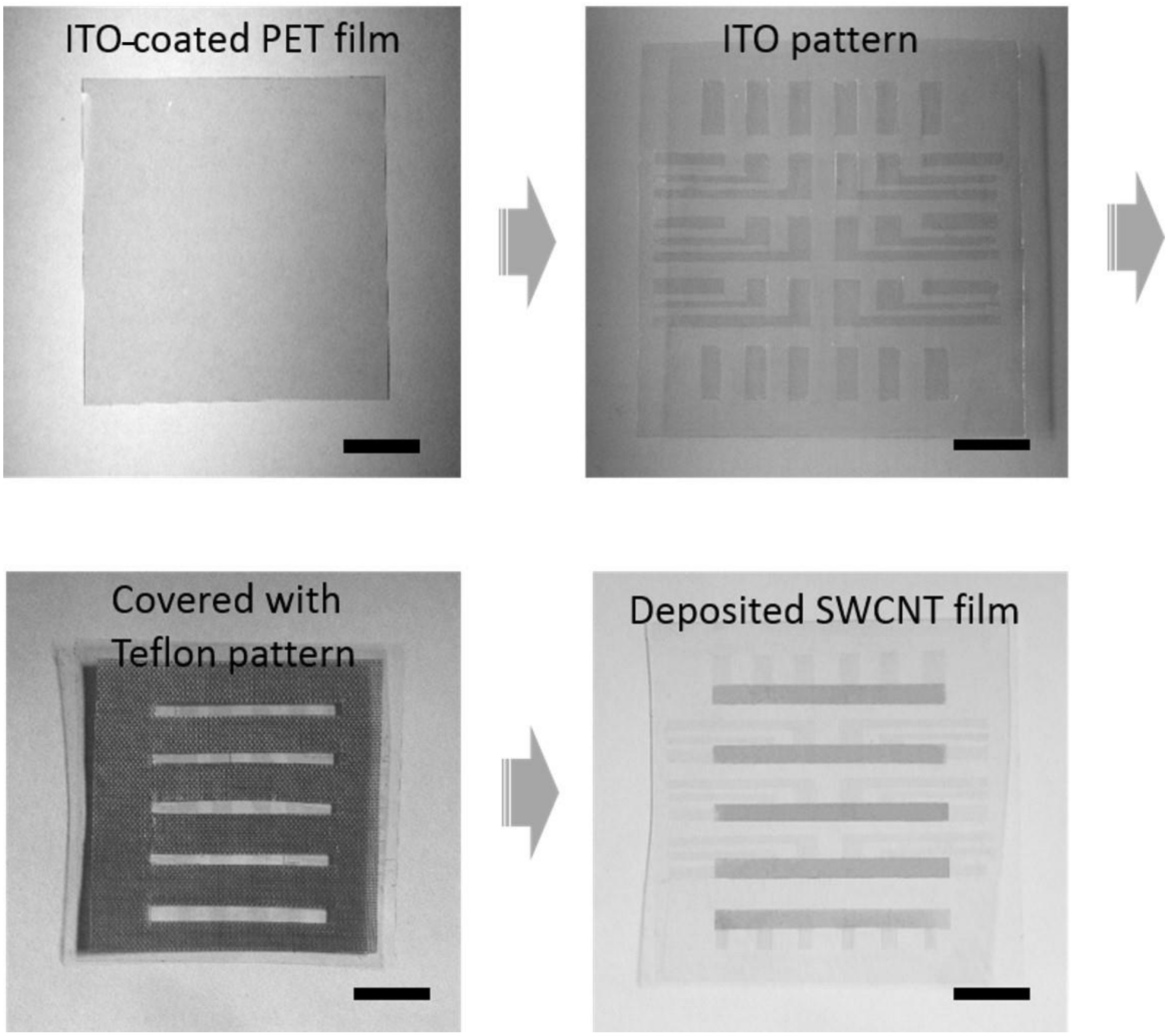

Figure S4. A typical fabrication process of the fully transparent, flexible, noncontact sensor panel with 5×5 sensing pixels. All scale bars are 1 cm.

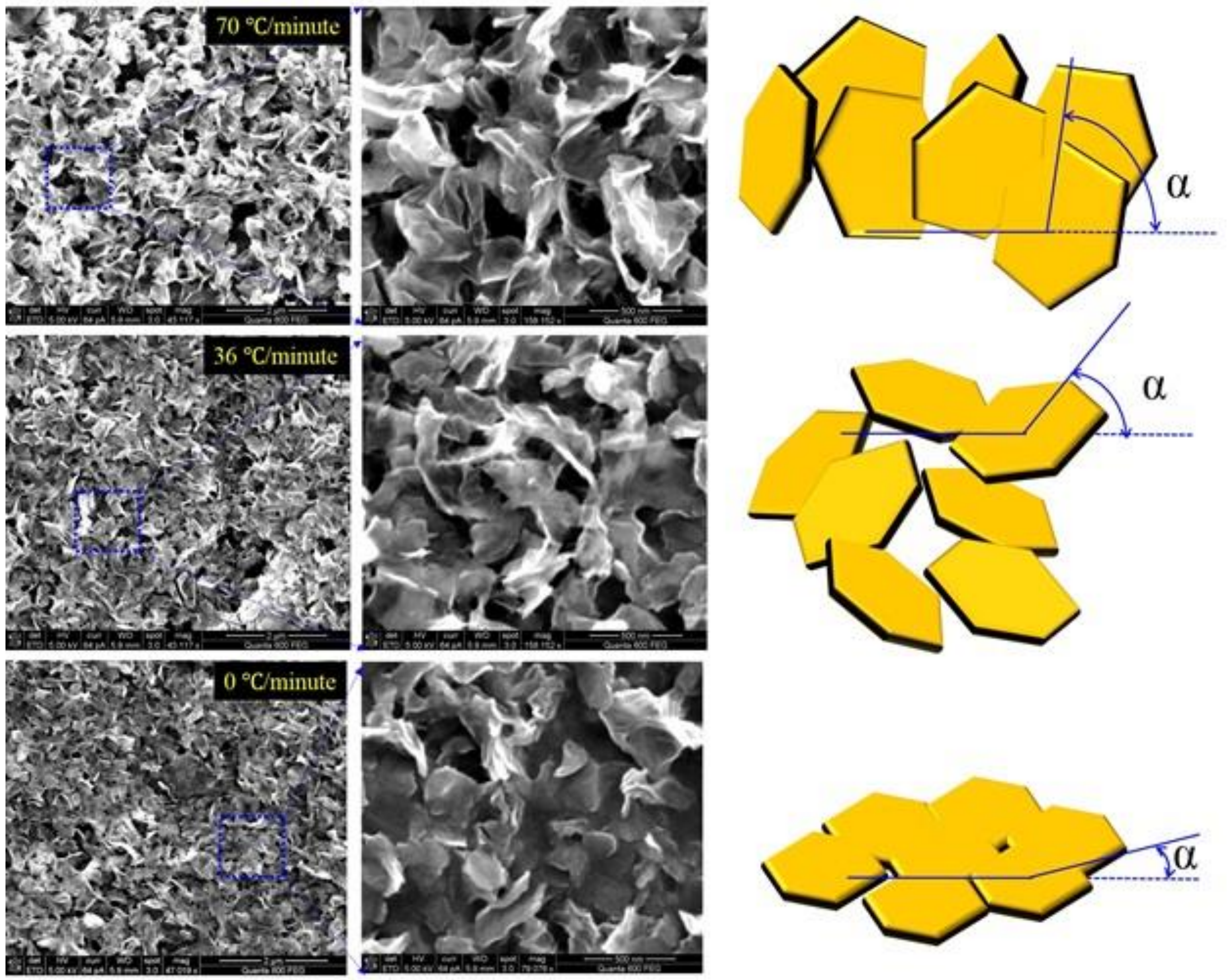

Figure S5. SEM images of graphene-PTCFs at different magnifications fabricated at different heating rates. Graphene concentration is 0.1 mg/ml, and the ratio of graphene to PEDOT:PSS is 1:0.5. (S2)

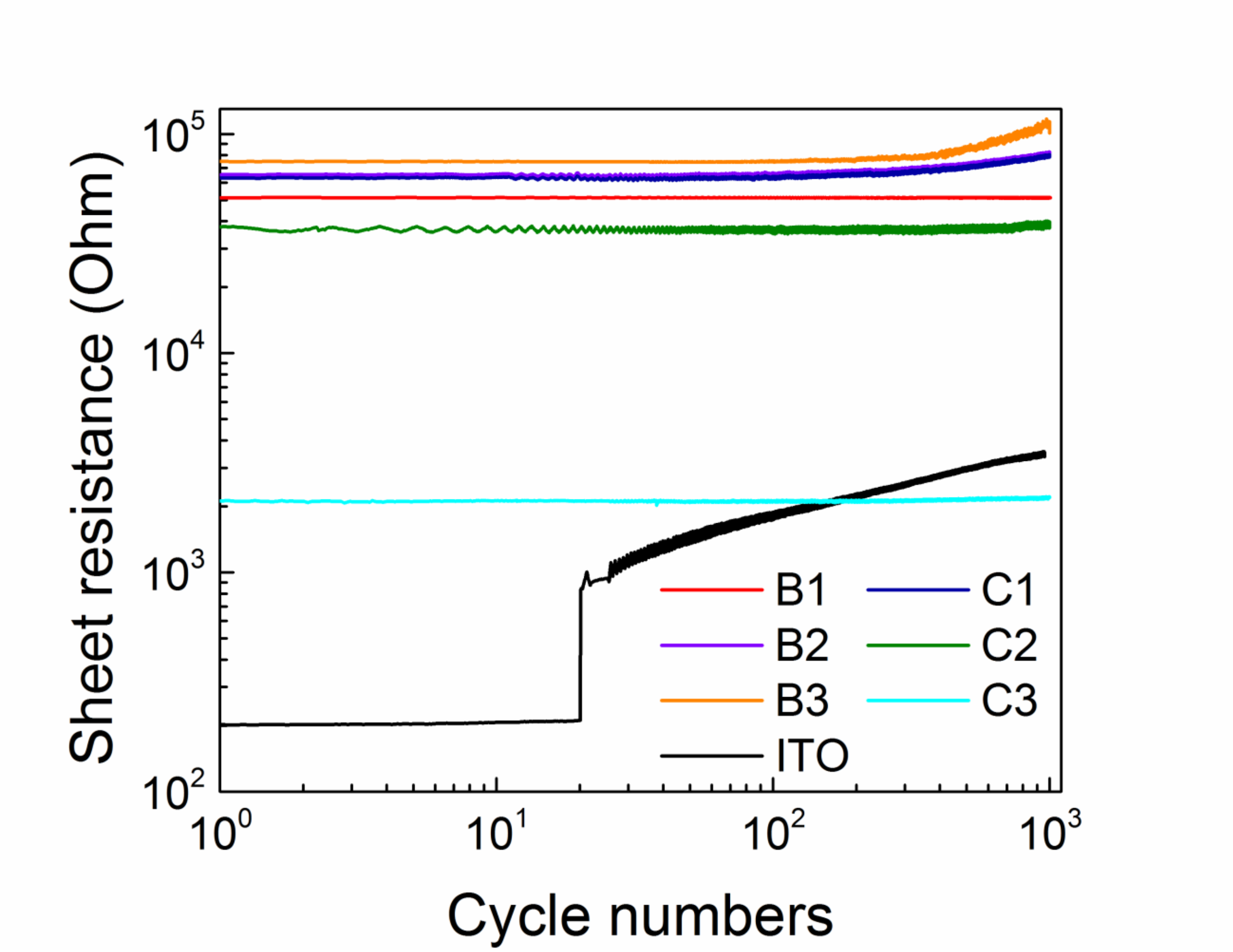

Figure S6. Properties of the mechanical resistance response of SWCNT-PTCFs (long cyclic tests under a dynamic force of 0.1 N with a preload of 0.1 N).

REFERENCES

S1. J. Han, B. Kim, J. Li, M. Meyyappan. Carbon Nanotube Based Humidity Sensor on Cellulose Paper. J. Phys. Chem. C 2012, 116, 22094−22097

S2. A. Zahab, L. Spina, P. Poncharal, C. Marliere, Water–vapor effect on the electrical conductivity of a single-walled carbon nanotube mat, Phys. Rev. B 2000, 62, 10000–10003
